# Role for the Mammalian Swi5-Sfr1 Complex in DNA Strand Break Repair through Homologous Recombination

**DOI:** 10.1371/journal.pgen.1001160

**Published:** 2010-10-14

**Authors:** Yufuko Akamatsu, Maria Jasin

**Affiliations:** Developmental Biology Program, Memorial Sloan-Kettering Cancer Center, New York, New York, United States of America; Brandeis University, United States of America

## Abstract

In fission yeast, the Swi5-Sfr1 complex plays an important role in homologous recombination (HR), a pathway crucial for the maintenance of genomic integrity. Here we identify and characterize mammalian Swi5 and Sfr1 homologues. Mouse Swi5 and Sfr1 are nuclear proteins that form a complex *in vivo* and *in vitro*. Swi5 interacts *in vitro* with Rad51, the DNA strand-exchange protein which functions during HR. By generating *Swi5* ^−/−^ and *Sfr1* ^−/−^ embryonic stem cell lines, we found that both proteins are mutually interdependent for their stability. Importantly, the Swi5-Sfr1 complex plays a role in HR when Rad51 function is perturbed *in vivo* by expression of a BRC peptide from BRCA2. *Swi5* ^−/−^ and *Sfr1* ^−/−^ cells are selectively sensitive to agents that cause DNA strand breaks, in particular ionizing radiation, camptothecin, and the Parp inhibitor olaparib. Consistent with a role in HR, sister chromatid exchange induced by Parp inhibition is attenuated in *Swi5* ^−/−^ and *Sfr1* ^−/−^ cells, and chromosome aberrations are increased. Thus, Swi5-Sfr1 is a newly identified complex required for genomic integrity in mammalian cells with a specific role in the repair of DNA strand breaks.

## Author Summary

Our genome constantly undergoes DNA damage as a result of agents in the environment, as well as from metabolic processes. One method of repairing DNA damage is homologous recombination (HR), in which genetic information from a duplicate sequence (the sister chromatid) is copied into the damaged site in DNA. In model organisms (the yeasts), a protein complex termed Swi5-Sfr1 functions in DNA damage repair by HR. In this study, we characterize mouse homologues of this complex. We find that mouse cells lacking this complex are sensitive to DNA damaging agents, in particular, those that cause breaks in DNA strands and that serve as cancer chemotherapeutics. These cells also have increased numbers of chromosome aberrations when exposed to DNA damaging agents. Moreover, HR is decreased in Swi5 and Sfr1 mutant cells under conditions where the cell is challenged. Together, these results demonstrate a requirement for the Swi5-Sfr1 protein complex in maintaining genomic integrity in mammalian cells.

## Introduction

Homologous recombination (HR) is a key pathway in mammalian cells for the repair of several types of lesions, including DNA strand breaks. Its importance is emphasized by the sensitivity of HR mutants to a variety of DNA damaging agents, as well as the loss of genomic integrity seen in these mutants arising from DNA damage. As a result, HR is a critical DNA repair pathway during development and for tumor suppression [1], [2].

Double-strand breaks (DSBs) arise in DNA as a result of both endogenous cellular processes and from exogenous sources [3], [4]. HR is a precise pathway for the repair of DSBs, during which homologous sequence information is copied from an intact donor template [1], [2], most frequently the sister chromatid during late S/G2 in mitotic cells [5]. A second key pathway for the repair of DSBs is nonhomologous end-joining (NHEJ), where two ends are joined with little or no sequence identity [6]. In addition to canonical two-ended DSBs, one-ended DSBs also arise in DNA [7]. These lesions form when a replication fork encounters a DNA single-strand break that is not repaired by base excision repair, for example, from a covalent topoisomerase I-DNA intermediate as a result of exposure to camptothecin [8], [9]. HR is the primary mechanism for the repair of one-ended DSBs, given that the joining of two unrelated one-ended DSBs by NHEJ would give rise to genomic rearrangements [7].

Many of the known HR factors in mammalian cells, including the central Rad51 protein, have been identified by their homology to yeast HR factors [2], [10], [11]. Rad51, the eukaryotic homologue of *Eschericia coli* RecA, binds to single-stranded DNA to form a nucleoprotein filament which catalyzes base pairing and strand exchange between homologous DNAs [12]–[14]. Single-stranded DNA is formed at DNA ends by resection [15]; although a substrate for Rad51 filament formation, single-stranded DNA is also bound by replication factor A (RPA), which binds at high affinity and removes secondary structure [16], [17]. While critical for the initiation of HR [18], RPA interferes with Rad51 loading onto single-stranded DNA. Several factors, referred to as “mediators”, are required to overcome the inhibition by RPA to facilitate Rad51 nucleoprotein filament formation [19]. Proposed mediators in yeast include the Rad51 paralogues, Rad55-Rad57, and Rad52 [20], [21]. Vertebrates have five Rad51 paralogues, of which a complex of two have been shown to have mediator activity *in vitro* [22]. Additionally, the breast cancer suppressor BRCA2, for which there is no homologue in budding or fission yeast, has been proposed to have mediator activity [23]. BRCA2 may also function to stabilize Rad51 filaments on single-stranded DNA, by inhibiting ATP hydrolysis while preventing the formation of non-productive filaments on double-stranded DNA [24].

A distinct complex that functions in fission yeast HR is Swi5-Sfr1. Mutation of either Swi5 or Sfr1 results in reduced HR in both mitotic and meiotic cells [25]–[28]. Like other HR mutants, Swi5 and Sfr1 mutants have elevated sensitivity to a number of DNA damaging agents, including ionizing radiation, UV, and methyl-methanesulfonate [29]. *In vitro*, the Swi5-Sfr1 complex binds to Rhp51 (the fission yeast Rad51 homologue) in an Sfr1-dependent manner [29], [30], and has been shown to possess mediator activity but importantly also to enhance strand exchange by Rhp51 [30]. While loss of either Swi5-Sfr1 or Rhp55-Rhp57 (fission yeast Rad55-Rad57 homologues) reduces HR, loss of both complexes complete abrogates Rhp51-dependent HR [26]. Both complexes are also required during meiotic recombination [31]. Budding yeast has a homologous complex to Swi5-Sfr1 termed Sae3-Mei5, although this complex is only expressed during meiosis where it plays a critical role in meiotic recombination [32]–[34].

Swi5 forms a second complex with an Sfr1-related protein, Swi2, which localizes to heterochromatin at the donor mating-type loci and promotes HR during switching [26], [29], [35]. In budding yeast, the function of Sae3-Mei5 appears to be limited to supporting the function of Dmc1, the meiosis-specific RecA homologue [33], [34].

Previous reports suggest that both Swi5/Sae3 and Sfr1/Mei5 are evolutionarily conserved, while Swi2 is only found in fission yeast [29], [34]. In this study, we isolated Swi5 and Sfr1 homologues from mice. Swi5 and Sfr1 form a complex *in vivo* and *in vitro*, and Rad51 binding to Swi5 is detected *in vitro* in GST-pull down assays, suggesting that the Swi5-Sfr1 complex has a conserved function in mouse. To investigate their function *in vivo*, we generated *Swi5^−/−^* and *Sfr1^−/−^* mouse embryonic stem (ES) cell lines. Although loss of either Swi5 or Sfr1 did not decrease HR frequency by itself, HR was perturbed to a greater extent in these cells by expression of a BRC peptide from BRCA2. Interestingly, *Swi5^−/−^* and *Sfr1^−/−^* cells were sensitive to ionizing radiation, camptothecin, and an inhibitor of poly(ADP-ribose) polymerase (Parp), all of which cause strand breaks. The induction of sister chromatid exchanges (SCE) by Parp inhibition was attenuated in the Swi5 and Sfr1-deficient cell lines; moreover, Parp inhibition resulted in increased chromatid breaks and radial chromosomes in *Swi5^−/−^* and *Sfr1^−/−^* cells. Thus, Swi5 and Sfr1 have an important role in the maintenance of genomic integrity in mammalian cells, in particular in the repair of DNA strand breaks.

## Results

### Cloning and structure of mammalian Swi5 and Sfr1

**Figure 1:**
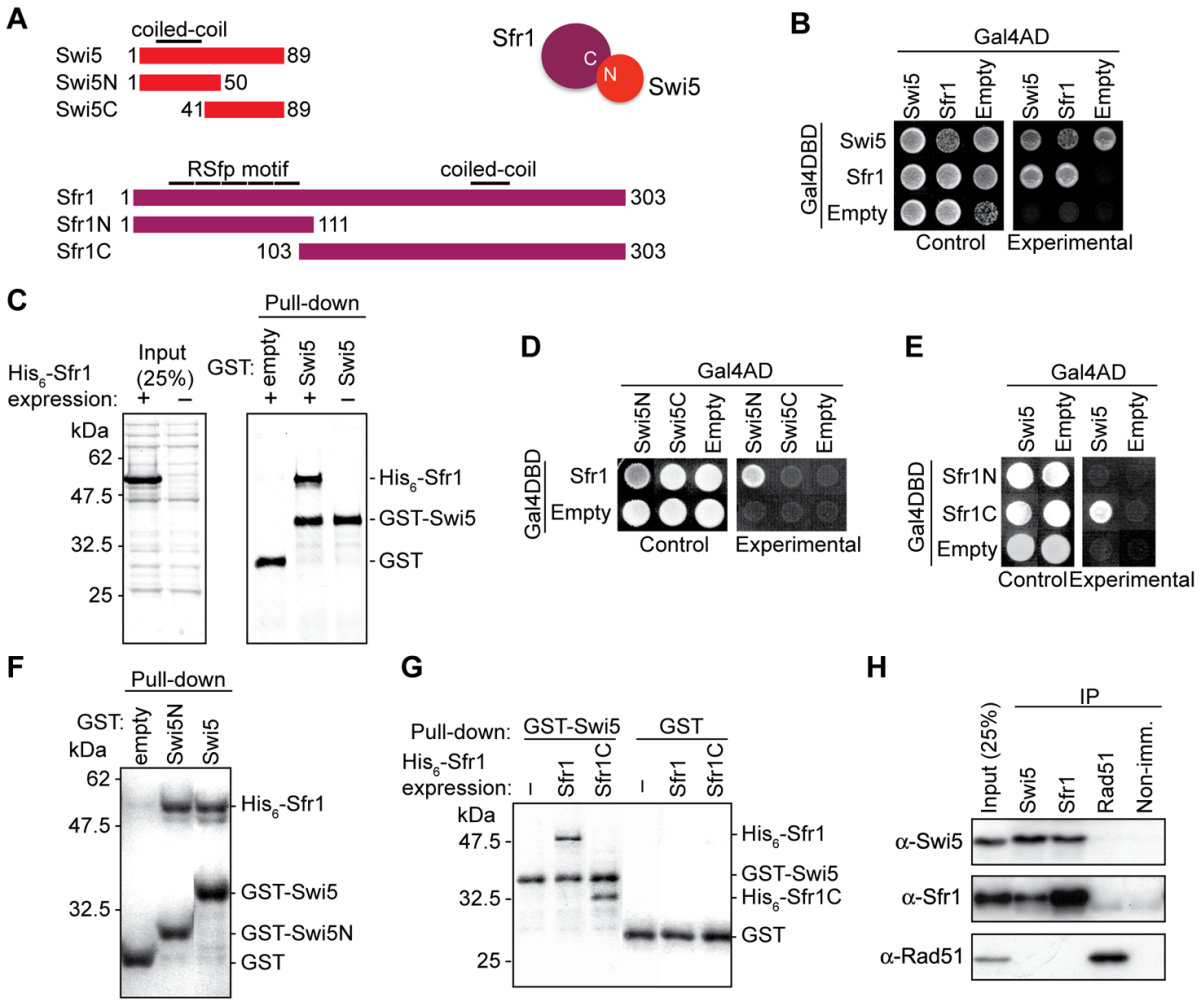
Mouse Swi5 and Sfr1 interact *in vivo* and *in vitro*. (A) Schematic diagrams depict Swi5, Sfr1 and the respective truncation mutants. (B) Interaction between Swi5 and Sfr1 was analyzed by yeast two-hybrid analysis. Reciprocal combinations of Gal4AD and Gal4DBD-fusion proteins were examined. Control plate, SD media without leucine and tryptophan; Experimental, SD media without leucine, tryptophan, histidine, and adenine. (C) Interaction between Swi5 and Sfr1 was analyzed by GST pull-down assays using recombinant proteins expressed in *E. coli*. CBB-stained SDS polyacrylamide gels of His_6_-Sfr1 expression (left) and pull-down by GST or GST-Swi5 fusion protein (right). (D) Yeast two-hybrid assay using the truncated Swi5 protein described in A. Gal4AD-Swi5N exhibited interaction with Gal4DBD-Sfr1. (E) Yeast two-hybrid assay using the truncated Sfr1 proteins described in A. Gal4DBD-Sfr1C interacted with Gal4AD-Swi5. (F) GST pull-down assay using Swi5N. Pull-down of GST-Swi5N precipitated His_6_-Sfr1. (G) GST pull-down assay using Sfr1C. Pull-down of GST-Swi5 precipitated His_6_-Sfr1C. (H) Swi5 and Sfr1 interaction in mouse ES cells was shown by co-immunoprecipitation using anti-Swi5 and anti-Sfr1 antibodies. Interaction with Rad51 was not observed in this assay.

Based on amino acid conservation, putative mammalian homologues of Swi5/Sae3 and Sfr1/Mei5 have previously been reported [29],[34]. We cloned the mouse homologues, 2900010J23Rik for Swi5 and 6330577E15Rik for Sfr1, based on existing database information (http://www.informatics.jax.org/ and http://uswest.ensembl.org/index.html). The Sfr1 cDNA was successfully amplified by PCR following reverse transcription (RT-PCR) of RNA obtained from mouse ES cells. Sequence analysis of the Sfr1 cDNA revealed that the Sfr1 protein is 303 amino acids and is encoded by four exons (Figure 1A, Figure S1A and S1B). Ectopic expression of the cloned cDNAs complemented the phenotypes of *Sfr1^−/−^*cell lines (see below).

Swi5 cDNAs were also obtained by RT-PCR of RNA from mouse ES cells. Two differentially spliced forms were detected containing alternative first exons which encoded proteins of 89 and 121 amino acids (Figure S2A and S2C). When expressed in ES cells, we found that both forms migrated at a lower molecular weight than the endogenous protein (Figure S2B); attempts to clone a cDNA expressing a larger protein were unsuccessful, possibly because the 5′ end of the mRNA contains a structure which impedes amplification or a non-AUG initiation codon. Nevertheless, both forms complemented the phenotypes of *Swi5^−/−^* cells (see below and data not shown). In subsequent experiments, we used the Swi5 cDNA encoding the 89 amino acid protein (Figure 1A).

Overall, the sequence identities between mouse and fission yeast proteins were 28.6% (Swi5) and 20.9% (Sfr1). Significant variation was noted between the N-terminus of the various Sfr1 orthologues, even among mouse strains. Mouse Sfr1 has a proline-rich repeat of 16 amino acids at its N-terminus, which we named the RSfp motif (rodent Sfr1 proline rich motif) (Figure 1A and Figure S1B). In the mouse ES cells used in this study (E14) and in DBA/2J mice (Q3TI03), there are five repeats of the RSfp motif, whereas in C57BL/6J mice there are six repeats. The rat Sfr1 homologue (rCG57555) has two repeats. Repetition of the RSfp motif appears to be unique to rodents, as only a single RSfp motif is present in other mammals, including human, rabbit, dog and pig (Figure S1C). The RSfp motif is not present outside of mammals, although the downstream region is conserved (Figure S1D).

### Mouse Swi5 and Sfr1 form a complex *in vivo* and *in vitro*

In fission yeast and in budding yeast, Swi5/Sae3 and Sfr1/Mei5 form a stable complex *in vivo* and *in vitro*. To determine whether mouse Swi5 and Sfr1 interact, we performed a yeast two-hybrid assay (Figure 1B). Swi5 fused to the Gal4 activation domain (AD) and Sfr1 fused to the Gal4 DNA binding domain (DBD) gave a positive interaction, suggesting a physical association between the two. The reverse test was uninformative as Swi5 fused to the Gal4DBD itself allowed growth on the test medium. In addition, Sfr1 showed self-association, which has also been observed in fission yeast [29].

**Figure 2:**
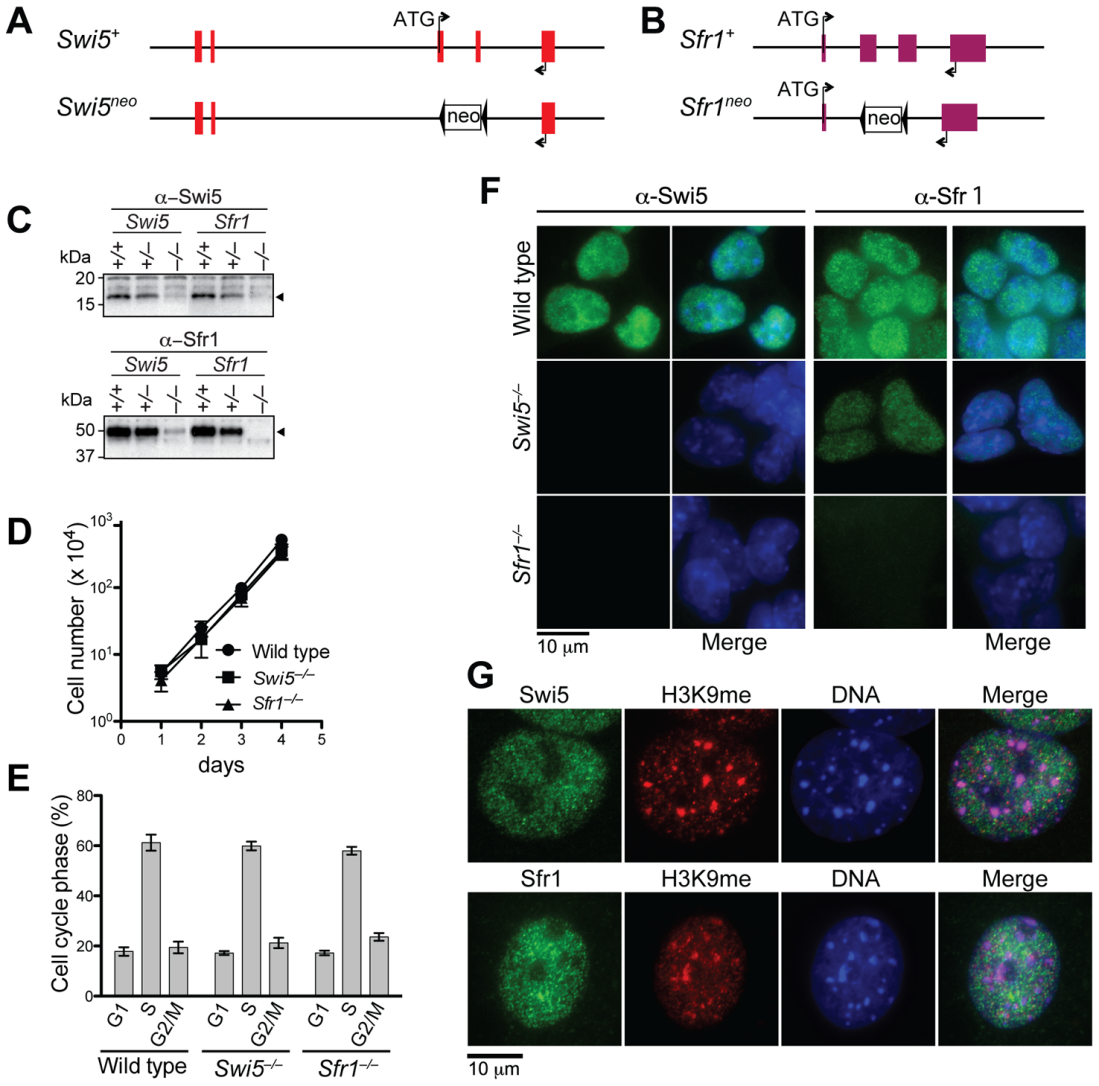
Swi5 and Sfr1 are mutually interdependent for their stability. (A) Schematic of genomic disruption of *Swi5*. Exons 3 and 4 are replaced by *neo*. (B) Schematic of genomic disruption of *Sfr1*. Exons 2 and 3 are replaced by *neo*. (C) Swi5 and Sfr1 expression was examined by Western blotting of mouse ES cell lines deficient for *Swi5* and *Sfr1*. The arrowheads indicate Swi5 and Sfr1 protein bands. There is a faint cross-reacting protein at the same position as Swi5. (D) *Swi5* ^−/−^ and *Sfr1* ^−/−^ cells proliferate with similar kinetics to wild-type cells. 2×10^4^ cells were seeded per well of a 6-well plate. Cell proliferation was measured by counting live cells at 24-hour intervals. Means with standard deviations (SD) are shown in the plot. (E) *Swi5* ^−/−^ and *Sfr1* ^−/−^ cells exhibit similar cell cycle profiles compared to wild-type cells. Propidium iodide stained cells were analyzed by flow cytometry. The cell cycle was calculated by FlowJo with a Watson pragmatic model. The means with SD are shown in the graph. (F) Localization of Swi5 and Sfr1 protein in mouse ES cells. Immunofluorescence of Swi5 and Sfr1 is shown in green in the respective left panels, with the DAPI-stained nucleus (blue) additionally shown in the merged images in the respective right panels. In the absence of Sfr1, Swi5 is not detectable; in the absence of Swi5, Sfr1 protein levels are reduced, but are still detectable. (G) Swi5 and Sfr1 do not show specific localization to heterochromatin detected by trimethyl-lysine 9 of histone H3 and intense DAPI staining in MEF cells.

We also tested complex formation with a GST pull-down assay using recombinant proteins expressed in *E. coli*. Unlike expression of yeast Sfr1/Mei5, which yields insoluble protein without co-expression of Swi5/Sae3 [30], , mouse His_6_-Sfr1 was soluble by itself (Figure 1C). The tagged Sfr1 migrated at a higher molecular weight (∼50 kDa) than the molecular weight calculated from the amino acid sequence (36 kDa). An unexpected lower mobility was seen with the endogenous Sfr1 protein (see below, Figure 2C). The *E. coli* extract expressing His_6_-Sfr1 was incubated with GST-Swi5 or GST alone immobilized on magnetic beads. Pull-down of GST-Swi5, but not GST, brought down His_6_-Sfr1 (Figure 1C), again indicating a physical association between Swi5 and Sfr1.

To determine their interacting domains, two-hybrid and GST pull-down assays were performed with N and C-terminal fragments from both proteins (Figure 1A). The N-terminal half of Swi5, but not the C-terminal half, interacted with Sfr1 in both assays (Figure 1D and 1F). Conversely, the C-terminal fragment of Sfr1, but not the N-terminal fragment, interacted with Swi5 (Figure 1E and 1G). The interacting fragments from both proteins contain coiled-coil motifs (Figure 1A), which may be responsible for the interaction. Consistent with their variability in different species, the RSfp motifs of Sfr1 did not appear to play a role in the interaction.

Co-immunoprecipitations were performed with mouse ES cell extracts to investigate the interaction *in vivo*. Using antibodies directed against the endogenous proteins, Swi5 co-precipitated Sfr1 and Sfr1 co-precipitated Swi5 (Figure 1H). Most of the Swi5 in the cell seems to be in a complex with Sfr1. Thus, despite the poor sequence conservation overall, Sfr1 is a major interacting partner for the Swi5 in the cell, consistent with the better conservation of the Sfr1 C-terminal portion, which interacts with Swi5.

### Swi5 and Sfr1 are nuclear proteins that are interdependent for protein stability

To investigate their cellular functions, we generated Swi5 and Sfr1-deficient mouse ES cell lines. The *Swi5* targeting vector was designed to replace the exons 3 and 4 with a *neomycin resistance* gene (*neo*), resulting in deletion of most of the Swi5 coding sequence, including the sequence for Sfr1 interaction (Figure 2A and Figure S3A). In the *Sfr1* targeting vector, the *neo* gene replaced exons 2 and 3, which encode amino acids 5 to 240, removing 78% of the coding region (Figure 2B and Figure S3B). Two rounds of gene targeting were performed, with an intervening step to delete the *neo* gene from the first targeted allele using Cre recombinase (Figure S3A and S3B). Successful gene targeting of both *Swi5* and *Sfr1* alleles in the respective cell lines was confirmed by Southern blotting (Figure S3A and S3B), and loss of protein was confirmed by Western blotting (Figure 2C) and immunofluorescence (Figure 2F). The *Swi5^−/−^* and *Sfr1^−/−^* cell lines (formally *Swi5^Δ/neo^* and *Sfr1^Δ/neo^*, respectively) exhibited similar proliferation kinetics and cell cycle distribution as wild-type cells (Figure 2D and 2E), indicating that Swi5 and Sfr1 are not essential for cell viability.

As Swi5 and Sfr1 form a complex, we determined whether loss of one affects the stability of the other by Western blotting (Figure 2C). Swi5 protein was not detectable in *Sfr1^−/−^* cells, indicating that the stability of Swi5 requires association with Sfr1. The level of Sfr1 in *Swi5^−/−^* cells was also diminished, although the protein was still detectable. These results provide further evidence for a physical association between Swi5 and Sfr1 *in vivo*.

To determine the sub-cellular localization of Swi5 and Sfr1, mouse ES cells and embryonic fibroblasts (MEFs) were examined by immunofluorescence. Swi5 and Sfr1 localized to the nucleus in both cell types (Figure 2F and 2G). Importantly, Swi5 was not detected in *Sfr1^−/−^* ES cells, providing further support that Swi5 is unstable without Sfr1; Sfr1 was detectable in *Swi5^−/−^* ES cells, albeit weakly (Figure 2F). In fission yeast, Swi5 localizes to heterochromatin as well as to euchromatin [26]. However, neither mouse protein specifically localized to heterochromatin, as marked by trimethyl-lysine 9 of histone H3 and intense DAPI staining (Figure 2G). Rather, both proteins had a more widespread nuclear distribution that was, nonetheless, somewhat granular.

### Mouse Swi5 interacts with Rad51

**Figure 3:**
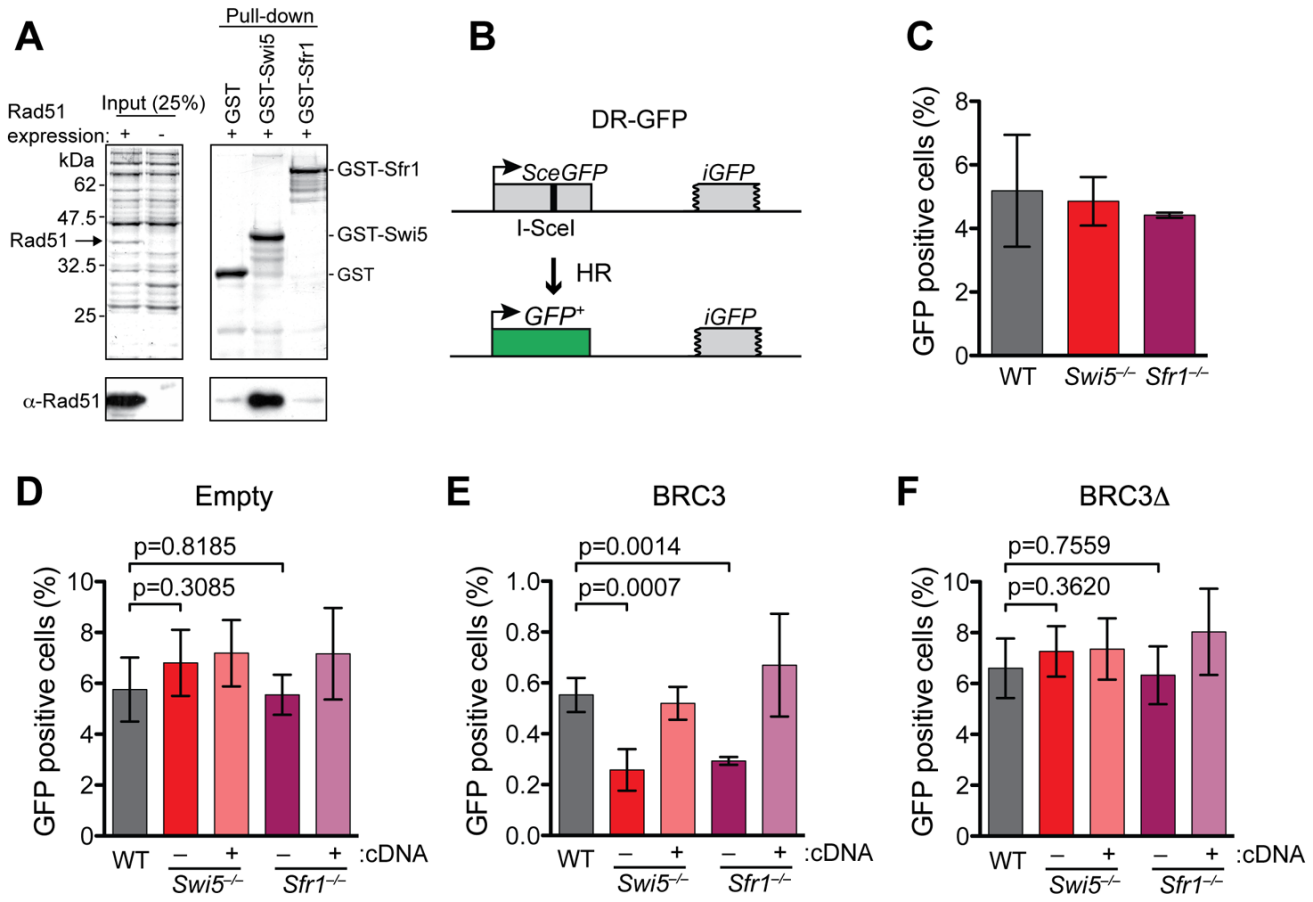
Swi5 and Sfr1 have a role in HR in mammalian cells. (A) Swi5 interacts with Rad51 in GST pull-down assay using recombinant proteins expressed in *E. coli*. The upper panels show CBB-stained SDS polyacrylamide gels. Note that Rad51 runs at similar molecular weight as GST-Swi5 (37 kDa). The pull-down was analyzed by Western blotting using anti-Rad51 antibody (EMD chemicals #PC130), as shown in the lower panels. (B) Schematic of the DR-GFP assay [37]. The DR-GFP construct consists of direct repeats of two mutated *GFP* genes, *SceGFP*, which is disrupted by an 18 bp recognition site for I-*Sce*I, and the truncated *iGFP*, genomically integrated into the *Hprt* locus. When a single DSB generated by I-*Sce*I is repaired via gene coversion with *iGFP*, expression of GFP is restored and can be measured by FACS analysis. (C) Unperturbed *Swi5* ^−/−^ and *Sfr1* ^−/−^ cells exhibit similar HR frequencies compared to wild-type cells after I-*Sce*I expression. (D–F) DR-GFP assays with co-transfection of empty, BRC3 or BRC3Δ expression vectors, respectively. *Swi5* ^−/−^ and *Sfr1* ^−/−^ cells show decreased HR with BRC3 expression. The respective cDNA expression constructs complemented the phenotypes of *Swi5* ^−/−^ and *Sfr1* ^−/−^ cells. Each value represents data from ≥3 independent experiments. Statistically significant differences are presented with p-values calculated using an unpaired t-test. Means with SD are shown in graphs in C–F.

The fission yeast Swi5-Sfr1 complex interacts with the Rad51 recombinase through the Sfr1 subunit [29], [30]. We tested whether Rad51 interaction would be conserved with the mouse proteins by co-immunoprecipitation from ES cell extracts. Neither Swi5 nor Sfr1 precipitated detectable amounts of Rad51 from either untreated (Figure 1H) or γ-irradiated cells (data not shown). To investigate this further, GST pull-down assays were performed with recombinant Rad51 expressed in *E. coli* (Figure 3A). Pull-down of Rad51 was detected with GST-Swi5, but not with GST-Sfr1 or GST alone (Figure 3A). Treatment of the extracts with ethidium bromide or DNase I did not affect the association between GST-Swi5 and Rad51 (Figure S4A). These results indicate a physical association between Swi5 and Rad51.

We tested whether Swi5 and Sfr1 co-localize with Rad51 in nuclear foci after X-irradiation. Unlike Rad51, Swi5 and Sfr1 were distributed throughout the nucleus, as in untreated cells, indicating that there was no specific recruitment of these proteins to DSB sites (Figure S4B). Further, Rad51 focus formation after X-irradiation was not noticeably affected in either *Swi5^−/−^* and *Sfr1^−/−^* cells (Figure S4C).

### HR is reduced in *Swi5^−/−^* and *Sfr1^−/−^* cells when HR is compromised

The conservation of the protein complex and the interaction with Rad51 suggested that Swi5-Sfr1 could play a role in HR in mammalian cells. We examined HR levels in the *Swi5^−/−^* and *Sfr1^−/−^* ES cells using the DR-GFP assay [37] (Figure 3B). In this assay, a single DSB is introduced into the chromosomally integrated DR-GFP substrate by the I-*Sce*I endonuclease; repair of the DSB by HR gives rise to cells expressing functional GFP. After I-*Sce*I expression, *Swi5^−/−^* and *Sfr1^−/−^* cells gave similar levels of GFP positive cells (4.9% and 4.4%, respectively) as wild-type cells (5.2%; Figure 3C), indicating that Swi5 and Sfr1 are not essential for HR in mouse cells.

In fission yeast, the Swi5-Sfr1 complex stabilizes Rad51 filament formation on single-stranded DNA [38]. We hypothesized that if Rad51 nucleoprotein filaments were perturbed in mouse cells, a role for the Swi5-Sfr1 complex in HR might be uncovered. BRCA2 is a central HR protein in mammalian cells, binding Rad51 at a series of repeats ∼35 amino acids (BRC repeats); as an isolated peptide, the BRC repeat has been demonstrated to bind Rad51, to inhibit Rad51 focus formation [39]–[41] and, importantly, to decrease HR in mammalian cells [42]. Compared to cells transfected with an empty expression vector (5.8%) (Figure 3D), expression of BRC3 in wild-type cells resulted in a significantly reduced frequency of GFP positive cells (0.55%) and hence HR (Figure 3E), consistent with previous results. This inhibitory effect on HR was not observed with expression of the BRC3Δ peptide which is unable to bind Rad51 [43] (6.6%; Figure 3F) and, further, was rescued by Rad51 overexpression (data not shown).

With BRC3 expression, *Swi5^−/−^* and *Sfr1^−/−^* cells exhibited a 2.1-fold and 1.9-fold reduction of GFP positive cells (0.26% and 0.29%, respectively) compared to wild-type cells (Figure 3E), indicating that Swi5-Sfr1 plays a role in HR when it is compromised. Consistent with this interpretation, expression of the cognate cDNAs complemented the HR defect (Figure 3E). The defect in HR was dependent on the ability of the BRC3 repeat to bind Rad51, as a similar number of GFP positive cells were obtained with BRC3Δ expression (Figure 3F). These results indicate that the Swi5 and Sfr1 function in HR, but are not required for the process unless it is already compromised. Because BRC3 perturbs Rad51 focus formation, mouse Swi5-Sfr1 may play a role in stabilizing Rad51 filaments, as in fission yeast.

### *Swi5^−/−^* and *Sfr1^−/−^* cells are sensitive to agents that cause strand breaks

**Figure 4:**
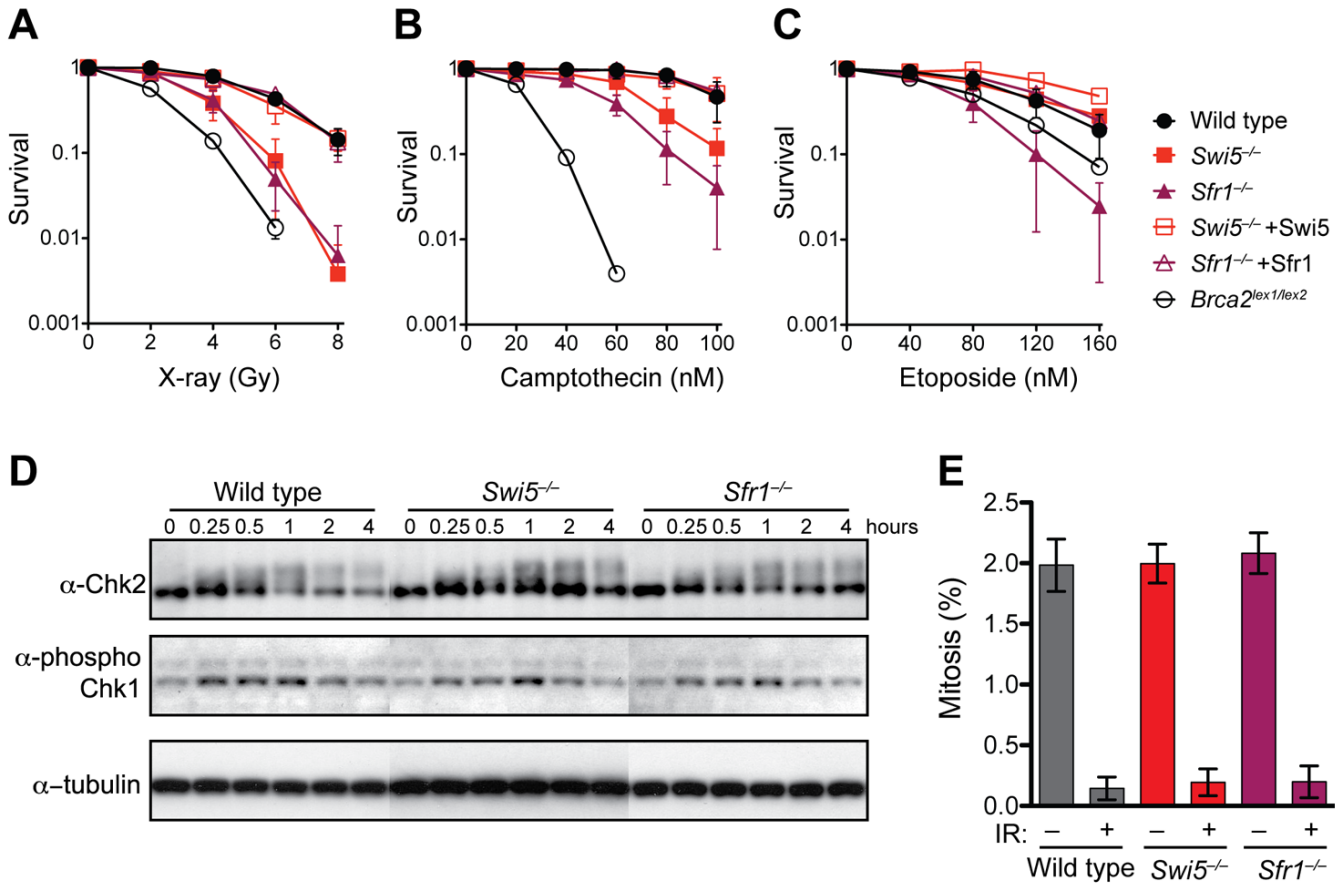
*Swi5* ^−/−^ and *Sfr1* ^−/−^ cells are defective in the repair of DNA strand breaks. (A–C) Clonogenic survival assays after treatment with X-rays, camptothecin and etoposide, respectively. (D) Normal induction of Chk2 and Chk1 phosphorylation by X-irradiation in *Swi5* ^−/−^ and *Sfr1* ^−/−^ cells. Cells irradiated with 8 Gy were collected at the indicated time points. Protein extracts were examined by Western blotting to analyze phosphorylation of Chk2 (Millipore #05-649) by mobility shift and Chk1 by a phospho-specific antibody against Serine 345 (Cell Signaling #2341). (E) Normal inhibition of mitotic entry in *Swi5* ^−/−^ and *Sfr1* ^−/−^ cells after X-irradiation. Cells irradiated at 8 Gy followed by two hours post incubation were fixed in 70% ethanol. Mitotic cells were stained using anti-phospho-histone H3 antibody (Millipore #06-570) and counted by flow cytometry. Means with SD are shown in graphs in A–C and E.

Given the HR phenotype associated with these cells, we next examined the sensitivity of *Swi5^−/−^* and *Sfr1^−/−^* cells to DNA damaging agents. In these assays, *Brca2^lex1/lex2^* cells were included for comparison, as they are known to be defective in HR [44]. *Swi5^−/−^* and *Sfr1^−/−^* cells were found to be more sensitive to X-rays than wild-type cells, although their sensitivity was less pronounced than that of *Brca2^lex1/lex2^* cells (Figure 4A). Expression of the Swi5 or Sfr1 cDNA in the respective mutant cells restored survival to the level observed in wild-type cells, demonstrating that the sensitivity was specifically due to the deletion of *Swi5* or *Sfr1*.

Cells were also exposed to topoisomerase poisons, which like X-rays lead to strand breaks. Both *Swi5^−/−^* and *Sfr1^−/−^* cells exhibited sensitivity to the type I topoisomerase poison camptothecin, although not as severely as *Brca2^lex1/lex2^* cells (Figure 4B). Interestingly, *Sfr1^−/−^* cells were somewhat more sensitive to camptothecin than *Swi5^−/−^* cells. *Sfr1^−/−^* cells were also sensitive to the type II topoisomerase poison etoposide. The two mutants again showed differential sensitivity, with *Sfr1^−/−^* cells showing a more severe phenotype. In this case, *Sfr1^−/−^* cells were even more sensitive than *Brca2^lex1/lex2^* cells, whereas *Swi5^−/−^* cells were no more sensitive than wild-type cells (Figure 4C). These results suggest that Swi5 and Sfr1 have a function in repairing DNA strand breaks, the primary lesions from X-irradiation and topoisomerase poisons. Given the greater sensitivity observed in *Sfr1^−/−^* cells, they also indicate that the roles of Swi5 and Sfr1 are not equivalent in the cell.

Cells with defective DNA damage checkpoints often exhibit sensitivity to DNA damaging agents. Chk1 and Chk2 are two proteins that are phosphorylated upon X-irradiation [45], [46]. After X-irradiation, *Swi5^−/−^* and *Sfr1^−/−^* cells were proficient at phosphorylation of both proteins and showed similar kinetics (Figure 4D). Checkpoint-proficient cells also arrest after DNA damage rather than proceed into mitosis. Mitotic populations were reduced to a similar extent in *Swi5^−/−^* and *Sfr1^−/−^* cells as in wild-type cells (Figure 4E). These results point to intact DNA damage checkpoints in both mutants.

We also tested the sensitivity of *Swi5^−/−^* and *Sfr1^−/−^* cells to a variety of other DNA damaging agents. HR mutants are typically sensitive to interstrand crosslinking agents [47]–[49], yet we observed that *Swi5^−/−^* and *Sfr1^−/−^* cells were not any more sensitive to either mitomycin C or cisplatin than wild-type or complemented cells (Figure S5A and S5B). In addition, cells were not sensitive to the replication inhibitor hydroxyurea (Figure S5C), implying that the camptothecin sensitivity is specifically related to strand breaks generated by this agent rather than indirectly to problems with replication per se. Finally, neither mutant was sensitive to ultraviolet light (Figure S5D), indicating that the proteins do not play a role in nucleotide excision repair. Interestingly, *Brca2^lex1/lex2^* cells were found to be sensitive, suggesting a role for HR repair of UVC lesions.

### *Swi5^−/−^* and *Sfr1^−/−^* cells are sensitive to Parp inhibition

**Figure 5:**
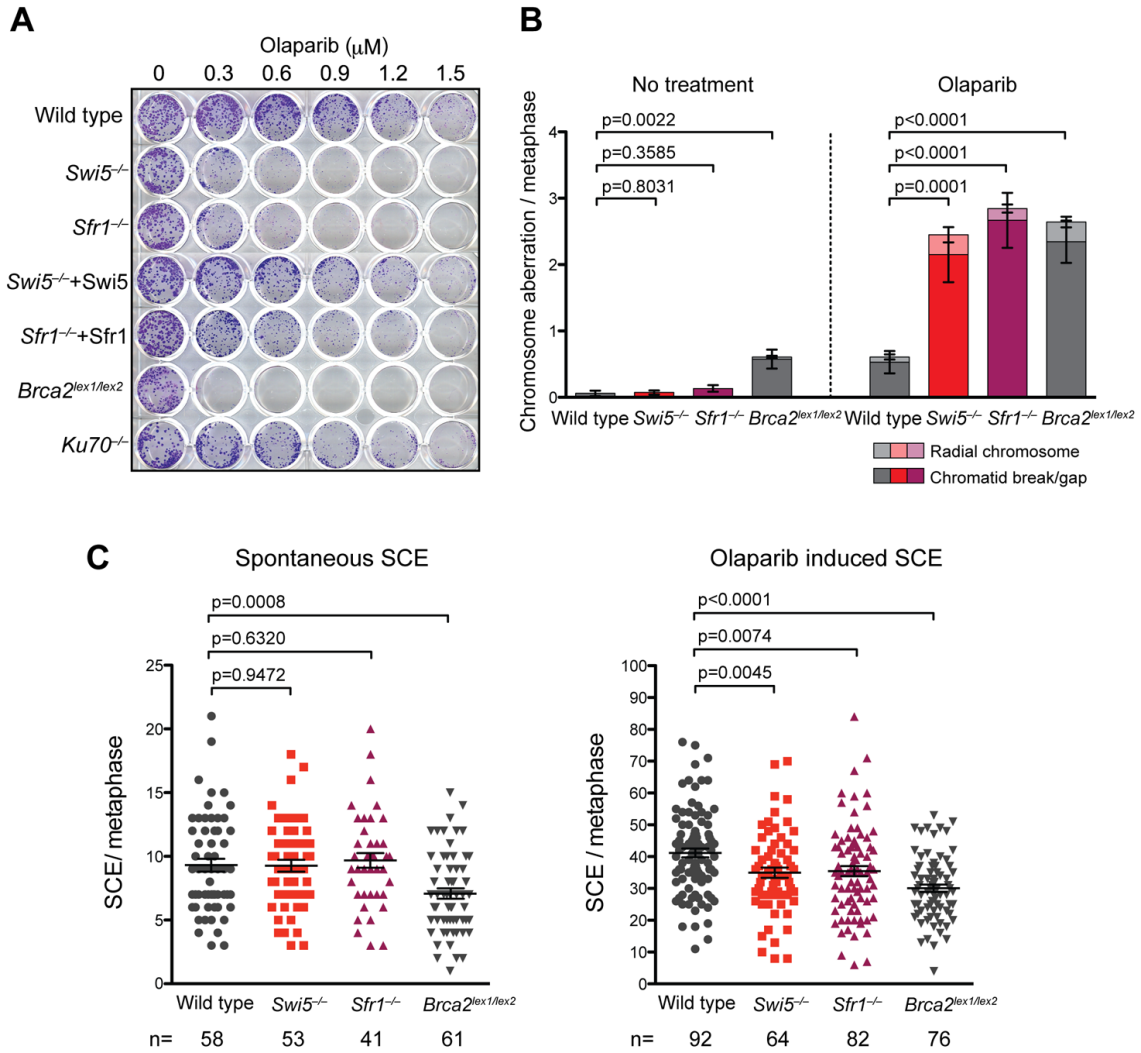
*Swi5* ^−/−^ and *Sfr1* ^−/−^ cells are sensitive to Parp inhibiton. (A) Hypersensitivity of *Swi5* ^−/−^ and *Sfr1* ^−/−^ cells to olaparib. Cells were grown with continuous exposure at the indicated concentration of olaparib. Surviving colonies were stained with Giemsa. (B) Chromosome aberrations are induced in *Swi5* ^−/−^ and *Sfr1* ^−/−^ cells to a greater extent than in wild-type cells after olaparib exposure. Metaphase spreads were prepared from cells with or without 48 hour exposure to 0.6 µM of olaparib. Chromosomes stained without banding using Giemsa were examined. Counts of chromosome aberrations are presented in Table S1. Means are shown with the standard error of the mean (SEM). The p-values were calculated using the Mann-Whitney test summing radial chromosomes and chromatid breaks/gaps. (C) Induction of SCEs by olaparib is lower in *Swi5* ^−/−^ and *Sfr1* ^−/−^ cells than in wild-type cells. The y-axis is the number of SCEs per metaphase for each nucleus counted. Cells were incubated in BrdU-containing medium for two cell cycles with or without exposure to 0.1 µM of olaparib for 6 hours before preparation of metaphase spreads. Indicated numbers of nuclei were counted. Means with SEM are shown in the plots. The p-values were calculated using unpaired t-test.

Poly(ADP-ribose) polymerase (Parp) plays an important role in the repair of DNA single-strand breaks, such that inhibition of Parp activity leads to the accumulation of the unrepaired single-strand breaks that turn into DSBs when encountered by replication forks. Since the repair of DSBs arising during replication largely depends on the HR pathway, cells deficient in HR are extremely sensitive to Parp inhibitors [50], [51]. To further investigate the effects of Swi5 and Sfr1 deficiency on the repair of DNA strand breaks, *Swi5^−/−^* and *Sfr1^−/−^* cells were exposed to the Parp inhibitor olaparib. Consistent with previous reports, *Brca2* mutant cells were exquisitely sensitive to olaparib; by contrast, the NHEJ mutant *Ku70^−/−^* was not (Figure 5A). *Swi5^−/−^* and *Sfr1^−/−^* cells were also significantly more sensitive to olaparib than wild-type cells, although not as sensitive as *Brca2^lex1/lex2^* cells (Figure 5A). This sensitivity was suppressed by introducing the cognate cDNAs into the *Swi5^−/−^* and *Sfr1^−/−^* cells (Figure 5A). Sensitivity of the cell lines to Parp inhibition further implicates Swi5 and Sfr1 in the repair of DNA strand breaks.

To further examine the effect of Parp inhibition on Swi5 and Sfr1-deficient cells, chromosomes were examined for aberrations in metaphase spreads. In *Swi5^−/−^* and *Sfr1^−/−^* cells, chromatid breaks were elevated 30 and 20-fold, respectively, after exposure to olaparib compared with untreated cells, significantly more than that observed in wild-type cells (9-fold; Figure 5B). Radial chromosomes, which were not observed in untreated cells, were also induced in *Swi5^−/−^* and *Sfr1^−/−^* cells. Both of these types of aberrations typically arise from problems encountered during DNA replication. *Brca2^lex1/lex2^* cells showed a substantial number of chromatid breaks even without olaparib, but chromatid breaks increased and radial chromosomes were observed upon olaparib treatment. The level of aberrations in olaparib-treated *Brca2^lex1/lex2^* cells was similar to that found in the treated *Swi5^−/−^* and *Sfr1^−/−^* cells, but aberrations may be underestimated if the G2/M checkpoint was activated. The observation of increased chromatid breaks and radial chromosomes in *Swi5^−/−^* and *Sfr1^−/−^* cells suggest that unrepaired DSBs accumulate, which may be responsible for the toxicity observed with Parp inhibition in these cells.

The accumulation of chromatid breaks induced by Parp inhibition may be the result of HR deficiency. To test this, we examined sister-chromatid exchange (SCE), which is one of outcome of HR (Figure 5C). The spontaneous SCE frequency was similar among wild-type, *Swi5^−/−^* and *Sfr1^−/−^* cells (9.3, 9.3 and 9.7 SCEs per metaphase, respectively), while *Brca2^lex1/lex2^* cells showed a lower frequency of SCE (7.1 SCEs per metaphase). With Parp inhibition, SCEs were significantly induced in wild-type cells (41.1 SCEs per metaphase) as well as in *Brca2^lex1/lex2^* cells, although the overall level was lower (30.1 SCEs per metaphase). In *Swi5^−/−^* and *Sfr1^−/−^* cells, the overall level of SCEs was reduced compared with wild type (35.0 and 35.4 SCEs per metaphase, respectively). These results indicate that SCE induction by Parp inhibition is partially dependent on Swi5 and Sfr1.

## Discussion

In this study, we identified Swi5 and Sfr1 orthologues in mammalian cells and determined that they have critical roles in the repair of DNA strand breaks. Despite their low conservation with the respective yeast proteins, we found that mouse Swi5 and Sfr1 form a complex *in vivo* and *in vitro*, as do fission yeast Swi5 and Sfr1 and budding yeast Sae3 and Mei5 [29], [30], [34]. The integral nature of the protein-protein interactions is emphasized by the mutual interdependence of the Swi5 and Sfr1 for stability, and by the finding that Sfr1 co-immunoprecipitates Swi5 to a similar extent as immunoprecipitation of Swi5 itself. Although the budding yeast complex is only expressed during meiosis [32], [34], mouse Swi5-Sfr1 is expressed in mitotically dividing cells, making it more akin to the fission yeast complex.

We found that Swi5 or Sfr1-deficient mammalian cells are sensitive to agents that cause DNA strand breaks, including X-rays, camptothecin, and the Parp inhibitor olaparib. Consistent with a DNA damage repair defect in *Swi5^−/−^* and *Sfr1^−/−^* cells, chromosome aberrations are increased compared to wild-type when cells are challenged with olaparib. For the most part, the sensitivities of *Swi5^−/−^* and *Sfr1^−/−^* cells are similar to each other, although unlike *Swi5^−/−^* cells, *Sfr1^−/−^* cells are also sensitive to etoposide. In contrast to Swi5, the stability of Sfr1 is not fully compromised when its partner protein is absent, consistent with Sfr1 functions that are independent of Swi5 in some contexts, as is the case with fission yeast [52], [53]. While fission yeast Swi5 acts independent of Sfr1 during mating-type switching, mouse Swi5 is unlikely to have Sfr1-independent functions, given its instability in the absence of Sfr1.

Sensitivity to camptothecin and olaparib is consistent with a defect in the ability to repair DNA damage by HR. That Swi5 interacts with Rad51 *in vitro*, the critical strand exchange protein for HR reactions, supports a role for the mammalian Swi5-Sfr1 complex in HR, like the cognate complexes in fission and budding yeast [26], [33], [34]. Further, DNA damage-induced SCEs are reduced in *Swi5^−/−^* and *Sfr1^−/−^* cells compared with wild-type cells. Moreover, although direct assay of DSB-induced HR in these cells did not reveal an intrinsic HR defect, a more severe defect in HR is observed in both the *Swi5^−/−^* and *Sfr1^−/−^* cells when HR is compromised by interfering with Rad51 function.

Unlike typical mammalian HR mutants, however, *Swi5^−/−^* and *Sfr1^−/−^* cells are not sensitive to interstrand crosslinking agents or the replication inhibitor hydroxyurea. Although both agents lead to DSBs during S phase and induce HR, DSBs are detected by pulse field gel electrophoresis only after prolonged incubation with these agents and require the structure-specific nuclease Mus81 for their formation [54], [55]. By contrast, when a replication fork encounters a single-strand break, a one-ended DSB is generated with fast kinetics, as DSBs appear within 30 min after camptothecin exposure during S phase [56]. In fission yeast, evidence points to a role for Swi5-Sfr1 (or Swi5-Swi2) acting specifically at one end of a DSB or at the one-ended DSBs at the *mat* locus during either mating-type switching or sister chromatid recombination in donorless strains [26], [57], [58]. Taken together, we propose that Swi5-Sfr1 is an evolutionarily conserved complex that acts at specific types of lesions, specifically at one-ended DSBs.

These experiments reveal a role for the mammalian Swi5-Sfr1 complex in HR. Although Swi5-Sfr1 are required for repair when the DNA damage load is high, the role of the complex appears to be more restricted than that of BRCA2 and the Rad51 paralogues, given the more severe phenotype seen when these other proteins are deficient [37], [44], [59]. In fission yeast, which does not have a BRCA2 orthologue, both Swi5-Sfr1 and the Rad51 paralogue complex Rhp55-Rhp57 are required for high levels of HR [26]. Thus, a shift in dependence on the Swi5-Sfr1 complex may have occurred during evolution. How might Swi5-Sfr1 function in HR? *In vitro*, the fission yeast Swi5-Sfr1 complex has mediator activity [30]. Moreover, the fission yeast Swi5-Sfr1 complex stabilizes the Rad51 filament on single-stranded DNA [38]. We hypothesize that the mammalian complex plays a similar role, given the reduced recombination in *Swi5^−/−^* and *Sfr1^−/−^* cells in the presence of the BRC3 repeat, which is known to perturb Rad51 focus formation [40], [41].

It is noteworthy, however, that the interaction of the Swi5-Sfr1 complex with Rad51 is through Swi5, in contrast to fission yeast where the interaction with Rhp51 is through Sfr1 [29], [30]. In both mouse cells and fission yeast, the interaction between Swi5-Sfr1 and Rad51 is detected *in vitro*, but not *in vivo*, as co-precipitation of the endogenous proteins has been unsuccessful, even under DNA damaging conditions [29]. Thus, Swi5-Sfr1 and Rad51 may interact weakly or transiently in cells. In fission and budding yeast, Swi5-Sfr1 and Sae3-Mei5, respectively, bind and promote the activity of Dmc1 [30], [33], [34], the meiosis-specific strand exchange protein, which is also critical for mouse meiosis [60], [61]. Whether Swi5-Sfr1 plays a similar role in mammalian cells awaits mouse knockout studies of the complex, although notably we have detected high level of expression of the complex in the testis, including a testis-specific isoform of Swi5 (Y.A. and M.J., unpublished results).

In summary, we have characterized a novel complex critical for DNA strand break repair in mammalian cells. The importance of strand break repair is well recognized, as defective repair is associated with various neurodegenerative diseases [62]. Moreover, therapeutic approaches to some cancers are being developed which increase the cellular load of DNA strand breaks through Parp inhibition [63]. The identification of Swi5-Sfr1 as being important for cellular resistance to agents like olaparib therefore has potential clinical as well as biological relevance.

## Materials and Methods

### Swi5 and Sfr1 cDNA cloning

Primers for Swi5 and Sfr1 cDNA cloning were designed based on annotated transcripts from the Ensembl database. For Swi5, forward-reverse primer pairs, YA110 (5′ATACCCACCCCTCCCAATAC)-YA113 (5′AGTTTAAGCCCACCCCACTC) and YA532 (5′ATTATTGTCGACATGGGAAGCAGGGGCGGAAC)-YA127 (5′GCCGGCGGCCGCTTACTATCAGTCATTCAGGTTTAGATC), were designed based on annotated transcripts ENSMUSG00000044627 and ENSMUST00000113400, respectively. For Sfr1, the forward-reverse primer pair, YA114 (5′GGCTGTGTGTACGGTGTGTC)-YA115 (5′CCTCCCTCTAAGCCACAACA), was designed based on annotated transcript ENSMUST00000099353. The genomic structures presented in Figures S1A and S2A were derived by comparing the amplified cDNA sequences to the genomic structures in Ensembl.

### GST-pull down assay

The full length and truncated Swi5 cDNAs were cloned into the GST expression vector pGEX6P-1 (GE healthcare). GST and GST-Swi5 proteins were expressed in *E. coli* (UT481). The cell lysates were obtained by sonication of cells in R-buffer (20 mM Tris-HCl, pH 7.6/1 mM EDTA/100 mM NaCl/0.1% Triton X-100/1 mM DTT/10% Glycerol) followed by centrifugation at 15000 ×g for 20 min. The expressed GST and GST-Swi5 protein in the lysates were immobilized to the MagneGST (Promega). The His_6_-Sfr1 and Rad51 proteins were expressed in *E. coli* BL21-CodonPlus (DE3) from plasmids pET15b or pET21d (Novagen) respectively. The lysates (40 µg of proteins) obtained in R-buffer with sonication followed by centrifugation were mixed with 10 µl of the GST or GST-Swi5 protein immobilized to MagneGST, and incubated three hours at 4°C. The precipitates were then washed three times with R- buffer and eluted by boiling in SDS-PAGE sample buffer. The co-precipitations were subjected to SDS-PAGE with Coomassie Brilliant Blue (CBB) staining and to Western blotting.

### Yeast two-hybrid assay

The full length and truncated Swi5 and Sfr1 cDNAs were cloned to pGADT7 or pGBKT7 expression vectors to fuse to the Gal4 activation domain (AD) or the Gal4 DBA-binding domain (DBD). The experiments were performed according to the manufacturer's instructions (Matchmaker Two-Hybrid system 3 from Clontech).

### Generation of mouse Swi5 and Sfr1 antibodies

The full length of Swi5 and Sfr1 cDNAs were cloned in pET15b vector (Novagen). The His_6_-tagged Swi5 and Sfr1 proteins, as immunogens, were expressed in *E. coli* (BL21-Codonplus DE3) and purified using affinity to TALON (Clontech). Polyclonal antisera against Swi5 and Sfr1 were generated by Covance. Each antiserum was affinity purified against the respective protein.

### Immunoprecipitataion

Protein extracts from mouse ES cells were obtained by lysing cells in L-buffer (50 mM Tris-HCl, pH 8.0/2 mM EDTA/125 mM NaCl/1% NP-40/Complete protease inhibitor cocktail from Roche/Halt phosphatase inhibitor mixture from Pierce) on ice for 20 min followed by centrifugation at 15000 ×g for 20 min. The antibodies were added to the protein extract (200 µg of protein) and then incubated for 2 hours at 4°C. Protein G Dynabeads (Invitrogen) were added and the mixtures were incubated for an additional hour. The precipitates were washed six times with W-buffer (50 mM Tris-HCl, pH 8.0/2 mM EDTA/200 mM NaCl/1% NP-40) and subsequently eluted by boiling in SDS-PAGE sample buffer. Immunoprecipitated proteins were analyzed by Western blotting.

### Immunofluorescence

The cytospin slide centrifuge was used to spread ES cells on glass slides. MEF cells were grown directly on cover slips. Cells were fixed with 4% paraformaldehyde in PBS for 10 min and then permeabilized in PBS containing 0.25% Triton X for 10 min. Following incubation with blocking buffer (10% FBS in PBS) for 1 hour, cells were incubated with the indicated primary antibodies (diluted in blocking buffer) for 16 hours at 4°C followed by Alexa 488 or 594 conjugated secondary antibodies (Invitrogen) for 2 hours at RT and washed with PBS before mounting in ProLong antifade reagent with DAPI (Invitrogen).

### Targeting vector construction

To create pYA163, the beta-actin promoter driven diphtheria toxin A (DTA) fragment from pBADT3-BSKII (a gift of Dr. Valter Agosti) and the *loxP-Neo-loxP* cassette from pEGFPKT1loxneo (a gift from Dr Willie Mark) was cloned into *Sma* I/*Xba* I and *Cla* I/*Hind* III sites respectively in pBluescript SK+. The targeting arms were PCR amplified from mouse genomic DNA, using primer sets; YA217 (5′TATAGTCGACTCTTTCCTTTCTCAGACATGGGTTC) and YA218 (5′ATATCTCGAGAACATTACAGATCAGAGTCTATGAATAT) for the *Swi5* long targeting arm; YA189 (5′GGTCTTGGAGTTTACTCCTTATC) and YA191 (5′GGCCCTCTGAAGATAAGATTTGT) for the *Swi5* short targeting arm; YA266 (5′ATAAACAATCAGCCAGATAACCAGA) and YA267 (5′TGAGACAGAAAGAGGGTGGATCT) for the *Sfr1* short targeting arm; YA270 (5′TAATGTCGACCATTTCCAACATCCAGCATTCCT) and YA271 (5′TATACTCGAGACGCGAATGATAATCAAATTATCTC) for the *Sfr1* long targeting arm. PCR products were confirmed by sequencing. The long targeting arms and the short targeting arms were cloned to pYA163 at *Sal* I and at *Eco* RV, respectively, to generate the *Swi5* targeting vector (pYA186) and the *Sfr1* targeting vector (pYA249).

### Cell lines

The E14 DR-GFP mouse ES cell line was established previously [37]. For gene targeting of Swi5 and Sfr1, 10 µg of *Sal* I-linearized targeting vectors were electroporated into 1×10^7^ E14 DR-GFP cells suspended in OPTI-MEM by pulsing cells at 250 V, 500 µF. After 24 hours of incubation, G418 was added at final concentration of 300 µg/ml. The medium was changed every other day. After 7 days, colonies were isolated and the gene targeting was confirmed by PCR and Southern blotting. To remove the *neo* gene, the pCAGGS-Cre vector (10 µg) was transiently transfected into cells by electroporation. Colonies were grown without G418 treatment, and clones were examined by PCR and Southern blotting. To create constructs which complement *Swi5* and *Sfr1* deficient cell lines, cDNAs encoding *Swi5* and *Sfr1* were cloned into the mammalian expression vector pCAGGS that was modified to contain a hygromycin resistance gene (*Hyg*) at *Hind* III. These constructs were electroporated into *Swi5^−/−^* and *Sfr1^−/−^* cells, and cells were grown in hygromycin to select stable clones. Swi5 and Sfr1 expressing cells were confirmed by Western blotting.

### DR-GFP assay

This assay has been described previously [64]. Briefly, 30 µg of I-*Sce*I expression vector, pCBASce was electroporated into ES cells suspended in 650 µl of OPTI-MEM (Invitrogen) at 250 V, 950 µF in a 0.4 cm cuvette. In the experiments with the BRC3 peptide, 30 µg of BRC3, BRC3Δ, or the empty expression vector [42] were additionally added. BRC3Δ contains a 7 amino acid deletion, abrogating the interaction with Rad51 [42], [43]. To measure HR frequency, GFP-positive cells were scored by flow cytometry at 48 hours following electroporation.

### Survival assay

For the clonogenic survival assay, 500 cells were seeded onto a 6 cm dish and incubated for 24 hours to allow cells to attach to the bottom. For X-ray sensitivity assays, cells were irradiated with the indicated doses. For camptothecin or etoposide sensitivity assays, cells were exposed to the indicated concentrations of drug continuously for 9 days. Then colonies were fixed with methanol and stained with Giemsa. To examine olaparib sensitivity, 500 cells were seeded per well of a 24-well plate. After 24 hours incubation, olaparib was added at the indicated concentration, and cells were continuously exposed to olaparib for 7 days before fixing and staining with Giemsa.

### Chromosome analysis

To prepare metaphase spreads, ES cells were treated with 0.03 µg/ml of colcemid for 30 min. Cells were collected and incubated in hypotonic solution (0.56% KCl) for 20 min. Subsequently cells were fixed in methanol: acetic acid (3∶1) and washed. The cell suspensions in fixative were spotted to slides and air-dried. To measure chromatid aberrations, the slides were stained with 2% Giemsa/Sorensen's buffer for 5 min. After washing with water, the spreads were mounted in Permount. To visualize SCE, ES cells were incubated with 10 µM BrdU during two cell cycles. After metaphase spreads were prepared, the slides were treated with 1 µg/ml Hoechst 33258 in Sorensen's buffer and rinsed with 2× SSC. The slides were exposed to black light for 20 min, incubated at 60°C for 2 hours, and stained in 2% Giemsa/Sorensen's buffer. The excess staining was washed with water and the slides were mounted in Permount.

## Supporting Information

Figure S1 Predicted mouse *Sfr1* open reading frame (orf) and sequence alignment of Sfr1 orthologues. (A) Structure of the *Sfr1* gene. (B) Predicted amino acid sequence of the *Sfr1* orf. Color differences represent individual exons. The repetitive RSfp motifs are located in exon 2 and the predicted coiled-coil motif is also indicated. The *Sfr1* gene disruption (see Figure 2B and Figure S3B) links exon 1 to exon 4, leading to a frameshift and creating a novel stop codon which is shown in the red box. (C) Alignment of the mouse Sfr1 N-terminal region with mammalian Sfr1 orthologues. Repetitive RSfp motifs are only found in rodents. (D) Alignment of the mouse Sfr1 C-terminus with eukaryotic Sfr1 orthologues. Sequence alignments were performed using ClustalX. Accession numbers for the Sfr1 orthologues are as follows: human (ENSP00000338089); pig (XP_001927262); dog (ENSCAFP00000015516); rabbit (ENSOCUP00000011010); rat (rCG57555); chicken (ENSGALP00000013642); frog (NP_001087482); fish (Zgc162162).
(1.07 MB PDF)

Figure S2 Alternative spliced forms of mouse *Swi5*. (A) Genomic structure of isolated *Swi5* cDNAs encoding 89 and 121 amino acid proteins. The difference in exon 1 usage results in an extended N-terminus for the 121 amino acid protein. (B) Comparison of ectopically expressed Swi5 proteins with endogenous Swi5 by Western blotting. *Swi5^−/−^* cells were transiently transfected with plasmids expressing either the 89 or 121 amino acid forms of Swi5. The cell extracts were prepared 24 hours after transfections. Both wild-type and *Swi5^−/−^* cells were transfected with empty vector as controls. (C) Predicted amino acid sequence of the *Swi5* orfs. Color differences represent individual exons. The predicted coiled-coil motif is indicated. The 121 amino acid spliced form of Swi5 has an additional 32 amino acids at the N-terminus which are shown in red.
(0.35 MB PDF)

Figure S3 Gene targeting of *Swi5* and *Sfr1*. (A) *Swi5* targeting strategy. An allele of *Swi5* was created by replacing exons 3 and 4 with a *loxP-neo-loxP* cassette to create the *Swi5^neo^*. Following deletion of *neo* by Cre recombinase, the second *Swi5* allele in the *Swi5* ^Δ*/+*^ cells was targeted to generate *Swi5^Δ/neo^* cells. Correctly targeted clones were confirmed by Southern blotting. The probes were designed outside of the targeting arm. (B) *Sfr1*targeting strategy. Exons 2 and 3 were replaced with the *loxP-neo-loxP* cassette. The *Sfr1^Δ/neo^* cell lines were obtained using the same procedure described for *Swi5^Δ/neo^*.
(0.45 MB PDF)

Figure S4 (A) Ethidium bromide (EtBr) and DNase I treatments did not interfere with co-precipitation of Rad51 by GST-Swi5 in the pull-down assay. During incubation of GST-Swi5 and Rad51 protein, 0.3 mg/ml of EtBr was added to the reaction. The DNase I treatment was performed against precipitates of GST or GST-Swi5 with 10 U of DNase I for 15 min at room temperature. After adding EDTA to stop the reaction, the precipitates were washed three times with TE and eluted with SDS-PAGE sample buffer. (B) Staining of Rad51 with Swi5 or Sfr1 in MEF cells observed 3 hours after 8 Gy of X-irradiation. (C) Rad51 focus formation was not affected in *Swi5^−/−^* and *Sfr1^−/−^* cells. The indicated mouse ES cell lines were exposed to 10 Gy of X-irradiation. Four hours post irradiation more than 90% of *Swi5^−/−^*and *Sfr1^−/−^* cells formed discrete Rad51 foci as similarly observed in wild-type cells. The merge images show co-staining with DAPI.
(9.18 MB TIF)

Figure S5 *Swi5^−/−^*and *Sfr1^−/−^* cells were not sensitive to mitomycin C (A), cisplatin (B), hydroxyurea (C) or UVC (D). Cells, seeded at 500 cells per well of a 24-well plate 24 hours earlier, were exposed to the reagents at the indicated concentrations, continuously for mitomycin C and hydroxyurea treatments and for 1 hour followed by a media change for cisplatin. Cells irradiated with UVC were seeded at 5000 cells per well of a 6-well plate 24 hours in advance of exposure at the indicated dose. After 7 days of incubation, cells were fixed and stained with Giemsa.
(5.92 MB TIF)

Table S1 Chromosome aberrations with or without Parp inhibition (0.6 µM).
(0.05 MB PDF)
