# Supplementary material for: Role for the Mammalian Swi5-Sfr1 Complex in DNA Strand Break Repair through Homologous Recombination
Source: PLoS Genet. 2010 Oct 14;6(10):e1001160. doi: 10.1371/journal.pgen.1001160 (PMC2954829; doi:10.1371/journal.pgen.1001160)
Supplement: Figure S1 — Predicted mouse Sfr1 open reading frame (orf) and sequence alignment of Sfr1 orthologues. (A) Structure of the Sfr1 gene. (B) Predicted amino acid sequence of the Sfr1 orf. Color differences represent individual exons. The repetitive RSfp motifs are located in exon 2 and the predicted coiled-coil motif is also indicated. The Sfr1 gene disruption (see Figure 2B and Figure S3B) links exon 1 to exon 4, leading to a frameshift and creating a novel stop codon which is shown in the red box. (C) Alignment of the mouse Sfr1 N-terminal region with mammalian Sfr1 orthologues. Repetitive RSfp motifs are only found in rodents. (D) Alignment of the mouse Sfr1 C-terminus with eukaryotic Sfr1 orthologues. Sequence alignments were performed using ClustalX. Accession numbers for the Sfr1 orthologues are as follows: human (ENSP00000338089); pig (XP_001927262); dog (ENSCAFP00000015516); rabbit (ENSOCUP00000011010); rat (rCG57555); chicken (ENSGALP00000013642); frog (NP_001087482); fish (Zgc162162). (1.07 MB PDF) [file pgen.1001160.s001.pdf]

A

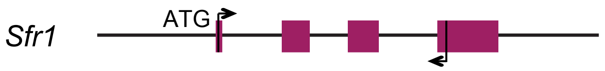

B *Sfr1* orf

```
ATG GCT GAG GAA GGA AAT CAG GAG TTC ACT TCC AAG ATG GAA AAC TCA TCA GAT TCA GCA
 M   A   E   E   G   N   Q   E   F   T   S   K   M   E   N   S   S   D   S   A
RSfp 1                                                             RSfp 2
TCG ACT TCA CCT GAT GCT CCT CAG CCC AGT GAG AAT CCA CCA TCA CCA CCG ACT TCA CCT
 S   T   S   P   D   A   P   Q   P   S   E   N   P   P   S   P   P   T   S   P
                                                RSfp 3
GCC GCT CCT CAG ACC AGT GAG AAT CCA CCA TCA CCA CCG ACT TCA CCT GCC GCT CCT CAG
 A   A   P   Q   T   S   E   N   P   P   S   P   P   T   S   P   A   A   P   Q
                                RSfp 4
CCC AGG GAG AAT CCA CCA TCA CCA CCG ACT TCA CCT GCC GCT CCT CAG CCC AGG GAG AAT
 P   R   E   N   P   P   S   P   P   T   S   P   A   A   P   Q   P   R   E   N
                RSfp 5
CCA CCA TCA CCA CCG ACT TCA CCT GCC GCT CCT CAG CCC AGG GAG AAT CCA CCA TCA CCC
 P   P   S   P   P   T   S   P   A   A   P   Q   P   R   E   N   P   P   S   P

CAT TCG AAT AGT TCA GGA AAA CAA CCT CTG AGT GGA ACA CCT AAA GAA AGG TTA AAG AAA
 H   S   N   S   S   G   K   Q   P   L   S   G   T   P   K   E   R   L   K   K

GCA AGA TCT TCA TCT CAT TCA TTT TGT AGT GTG GTG AAG CGT ATG AAG GTA GAG AAT GAC
 A   R   S   S   S   H   S   F   C   S   V   V   K   R   M   K   V   E   N   D

GAA AAT AAT GAG ACC CTT TCA GAA CCA GGA GAA TCT TCA AAG GAA GAA AAT TGT TCA AAG
 E   N   N   E   T   L   S   E   P   G   E   S   S   K   E   E   N   C   S   K

GCC CAA GAA AGC TTG AAA AAC AAA GAC AGT GAG CCT GGA GAA AAG AGC TCA GAG GAG AAA
 A   Q   E   S   L   K   N   K   D   S   E   P   G   E   K   S   S   E   E   K

AAC ACG TGT GAA TCT AAG TCG TCG GAT ACT GGG TCA TCT AAT GCC CTC CCA AAG GAG TCT
 N   T   C   E   S   K   S   S   D   T   G   S   S   N   A   L   P   K   E   S

GAG AAT GCG ATC ATT AGA GAA AAA TTA AAA CAA GAA AAA ATA AGA TTG ATT AGG CAG GTT
 E   N   A   I   I   R   E   K   L   K   Q   E   K   I   R   L   I   R   Q   V
                     Coiled-coil
GAA GAG AAG GAA GAC CTT CTT CGG AGG TTA AAG TTA GTC AAG ATG TAT AGA ATA AAG AAC
 E   E   K   E   D   L   L   R   R   L   K   L   V   K   M   Y   R   I   K   N

GAC GTG ACT GAG TTA GAG AAT CTA ATA AAG AAG TGG AGG AAG TGT GGT CAG CGG CTG CTG
 D   V   T   E   L   E   N   L   I   K   K   W   R   K   C   G   Q   R   L   L

TGT GAG CTG CAG TCT ATC ATG TCG GAG GAC GAG GAC GAG AAG CTG ACG CTC ACC GAG CTG
 C   E   L   Q   S   I   M   S   E   D   E   D   E   K   L   T   L   T   E   L

ATC GAC TTC TAC GGG ATA GAT GAC AAC TTG CTG CAC TAC AAT CGA AGT GAA GAA GAG TTT
 I   D   F   Y   G   I   D   D   N   L   L   H   Y   N   R   S   E   E   E   F

ACA GGG GTG TGA
 T   G   V   *
```

C Sfr1 N-ter alignment

```
  human   64 -SFLEKNQDFTFKMESPSDSAVVLPSTPQASANPSSP  99
    pig   78 MAAGEVNQD--FKMGSP-DSAVILPSTPQAGANPPSP  111
    dog   57 -NFVEVSQDCVFKMESPSDPAVVSPSTPQACASPPSP  92
 rabbit    1 FLVLEVTQDFIFKMESPSDSAVIAPSTPQACANPPSS  36
    rat  [ 1 MAEEEGNQEITSKMENPSDSASTSPDIPQTSENPPSP  37
         [                       38 PTSPDKPQTSENPPSL  53
  mouse  [ 1 MAEE-GNQEFTSKMENSSDSASTSPDAPQPSENPPSP  36
         [                       37 PTSPAAPQTSENPPSP  52
         [                       53 PTSPAAPQPRENPPSP  68
         [                       69 PTSPAAPQPRENPPSP  84
         [                       85 PTSPAAPQPRENPPSP  100
                                    ________________
                                       RSfp motif
```

D Sfr1 C-ter alignment

```
human          100 YTNSSRKQPMSATLRERLRKTRFSFNSSYNVVKRLKVESEENDQTFSEKPASSTEENCLEFQESFKHIDSEFEENTNLKN 179
rat             54 HSNSSGKQPMSGTLKERLKKARASSQPFCSVVKRIKVENEENDQTLSEPGESSKEENCSKAQESLENKDNEPEKES---- 129
mouse          101 HSNSSGKQPLSGTPKERLKKARSSSHSFCSVVKRMKVENDENNETLSEPGESSKEENCSKAQESLKNKDSEPGEKS---- 176
chicken         25 QRTSSGKQPMSATLRERLRKTRRSFTTNFAVAKRLKVDTEEKD-CTDVDNRCLPK---IGMDCSTLQDGSECLEQN---- 96
frog            23 -KDTVPKQPMSATLRERLRKTRRSFNAAFSVAKRLKVDCEENESSSDCPNNNLP-----------PHEDSEPNATK---- 86
fish            22 SNRSSTAKPMSASLREKLKRSRHSFKSPLSVVKRLKIEDDTEPQPSQQ---------------------GEEEKHG---- 76
S.pombe         72 TPDLRDTKIHTSLPITTPFSKKRAREAKNILLKPFKSPLRQTASPQVADTNLKPSLAVTNLNSDETNTSSEPVTSP---- 148
S.cerevisiae     1 ----------MHNQEEWLDKDK-------------TLVNEEENTCINHS------------------------------- 26

human          180 TLKNLNVCESQSLDSGSCSALQNEFVSEKLPKQRLNAEKAKLVKQVQEKEDLLRRLKLVKMYRSKNDLSQLQLLIKKWRS 259
rat            130 -SEDKNTSESKSLDTGSSSVLQKD-STEKTIKQTLKEEKAKLTRQVQEKEDLLRRLKLVKMYRIK--------------- 192
mouse          177 -SEEKNTCESKSSDTGSSNALPKE-SENAIIREKLKQEKIRLIRQVEEKEDLLRRLKLVKMYRIKNDVTELENLIKKWRK 254
chicken         97 -CTVNTYFKSPLQENNLCESAENVVRVDLG-QQQSLGEKVRLMKQVKEKEELLRRLKLVKMYRSKNNLSELQALIVKWRS 174
frog            87 -IEVDLVSEGDICRLDSRTSSEASSPVRCKDHKQLLQEKKLLLKKLEEQEDTLRRLKMVKLYRAKNNLSELQSLIEKWRK 165
fish            77 ---VKDDRDSNVTETDVNRNDMKLQRDSNHTAELPSQQCEALRKAVKERTETLRRLKMVKMYRKKNDLNELQRLTDKWRS 153
S.pombe        149 -LRTTPNSIKRQKRLFKSPISNCLNPKSDPEITQLLSRRLKLEKEVRNLQEQLITAETARKVEAKNEDKDLQTLIQKWKN 227
S.cerevisiae    27 -YTKKDTNNYRVGKSGIKDLKKPTNQKEIAIKNRELTKQLTLLRQENN------HLQQACKILSENKIIENRKSIEKWRT 99

human          260 CSQLLLYELQSAVSE-ENKKLSLTQLIDHYGLDDKLLHYNRSEEEFIDV 307
rat                -------------------------------------------------
mouse          255 CGQRLLCELQSIMSEDEDEKLTLTELIDFYGIDDNLLHYNRSEEEFTGV 303
chicken        175 STQLMLYELQAAFSA-DGKKVSLSQLIDTFGLEDHLLHYSRAEEDFVDT 223
frog           166 SSQLSLYELQAALIA-ENNKVTLSQLIESYGLDENLLHYNRTEEDFQDQ 213
fish           154 CAQSVLYELQRELAT-GGKQASLSQLIDSFGINDKLLHFDRTEEDFTDT 201
S.pombe        228 AAQQAAEVLFKPMAERIRLAGGVTQSFRIE-EGENKGQIQEVRTEFTMS 275
S.cerevisiae   100 ICEMELSFILNSTLIKINRMGGYKDFLEKEMEAKKRRLEYQIDNGMEDQ 148
```
