# Supplementary material for: Role for the Mammalian Swi5-Sfr1 Complex in DNA Strand Break Repair through Homologous Recombination
Source: PLoS Genet. 2010 Oct 14;6(10):e1001160. doi: 10.1371/journal.pgen.1001160 (PMC2954829; doi:10.1371/journal.pgen.1001160)
Supplement: Figure S2 — Alternative spliced forms of mouse Swi5. (A) Genomic structure of isolated Swi5 cDNAs encoding 89 and 121 amino acid proteins. The difference in exon 1 usage results in an extended N-terminus for the 121 amino acid protein. (B) Comparison of ectopically expressed Swi5 proteins with endogenous Swi5 by Western blotting. Swi5−/− cells were transiently transfected with plasmids expressing either the 89 or 121 amino acid forms of Swi5. The cell extracts were prepared 24 hours after transfections. Both wild-type and Swi5−/− cells were transfected with empty vector as controls. (C) Predicted amino acid sequence of the Swi5 orfs. Color differences represent individual exons. The predicted coiled-coil motif is indicated. The 121 amino acid spliced form of Swi5 has an additional 32 amino acids at the N-terminus which are shown in red. (0.35 MB PDF) [file pgen.1001160.s002.pdf]

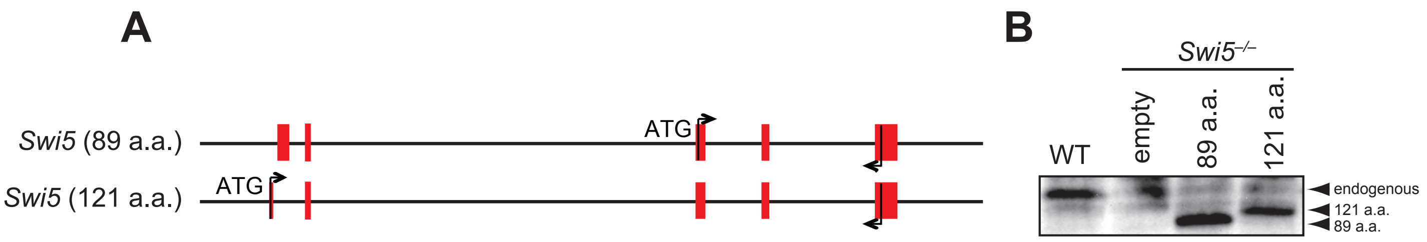

C

Swi5 orf (89 amino acids)

```
atg att gat gag aat aac gat gtc agc gag gag gcc ttg agc tct gac att aag aaa ctg
 M   I   D   E   N   N   D   V   S   E   E   A   L   S   S   D   I   K   K   L
                                       Coiled-coil
aag gag aag cat gac atg ctg gac aag gag atc tcc cag tta ata gca gag ggc tac cgt
 K   E   K   H   D   M   L   D   K   E   I   S   Q   L   I   A   E   G   Y   R

gtg att gag ctg gag aag cat atc tcc ctc ctc cat gag tac aat gac atc aag gat gta
 V   I   E   L   E   K   H   I   S   L   L   H   E   Y   N   D   I   K   D   V

tca cag atg ctg ctg ggg aag ctg gct gtg act cga ggc gtt acc acc aag gag tta tat
 S   Q   M   L   L   G   K   L   A   V   T   R   G   V   T   T   K   E   L   Y

cca gat ttt gat cta aac ctg aat gac tga
 P   D   F   D   L   N   L   N   D   *
```

Swi5 orf (121 amino acids)

```
atg gga agc agg ggc gga acc gct tta act tgg ggt gag tca gaa ttc agc cga ctt tac
 M   G   S   R   G   G   T   A   L   T   W   G   E   S   E   F   S   R   L   Y

cat ggc ggc tac cga tca ccg caa cgg cca ttc ccc atg att gat gag aat aac gat gtc
 H   G   G   Y   R   S   P   Q   R   P   F   P   M   I   D   E   N   N   D   V

agc gag gag gcc ttg agc tct gac att aag aaa ctg aag gag aag cat gac atg ctg gac
 S   E   E   A   L   S   S   D   I   K   K   L   K   E   K   H   D   M   L   D
      Coiled-coil
aag gag atc tcc cag tta ata gca gag ggc tac cgt gtg att gag ctg gag aag cat atc
 K   E   I   S   Q   L   I   A   E   G   Y   R   V   I   E   L   E   K   H   I

tcc ctc ctc cat gag tac aat gac atc aag gat gta tca cag atg ctg ctg ggg aag ctg
 S   L   L   H   E   Y   N   D   I   K   D   V   S   Q   M   L   L   G   K   L

gct gtg act cga ggc gtt acc acc aag gag tta tat cca gat ttt gat cta aac ctg aat
 A   V   T   R   G   V   T   T   K   E   L   Y   P   D   F   D   L   N   L   N

gac tga
 D   *
```
