# Supplementary figures and images for: Role for the Mammalian Swi5-Sfr1 Complex in DNA Strand Break Repair through Homologous Recombination
Source: PLoS Genet. 2010 Oct 14;6(10):e1001160. doi: 10.1371/journal.pgen.1001160 (PMC2954829; doi:10.1371/journal.pgen.1001160)

**A**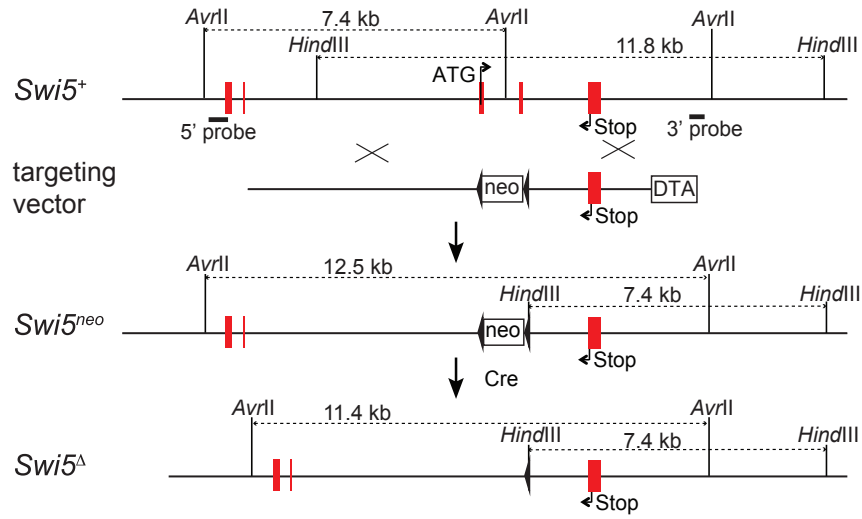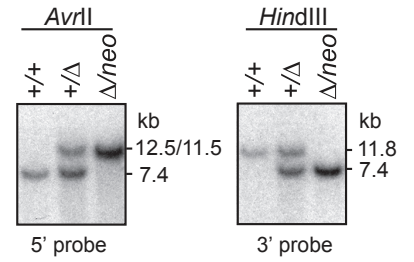**B**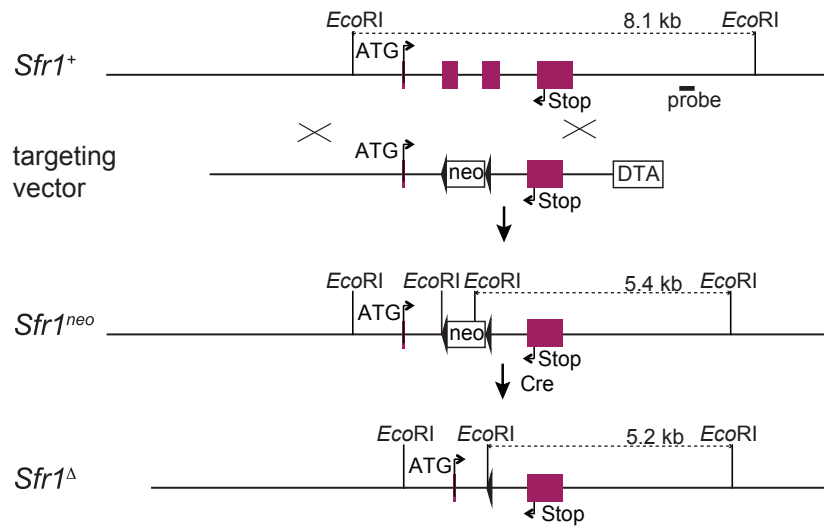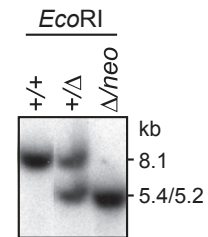

Supplement: Figure S3 — Gene targeting of Swi5 and Sfr1. (A) Swi5 targeting strategy. An allele of Swi5 was created by replacing exons 3 and 4 with a loxP-neo-loxP cassette to create the Swi5neo. Following deletion of neo by Cre recombinase, the second Swi5 allele in the Swi5 Δ/+ cells was targeted to generate Swi5Δ/neo cells. Correctly targeted clones were confirmed by Southern blotting. The probes were designed outside of the targeting arm. (B) Sfr1targeting strategy. Exons 2 and 3 were replaced with the loxP-neo-loxP cassette. The Sfr1Δ/neo cell lines were obtained using the same procedure described for Swi5Δ/neo. (0.45 MB PDF) [file pgen.1001160.s003.pdf]

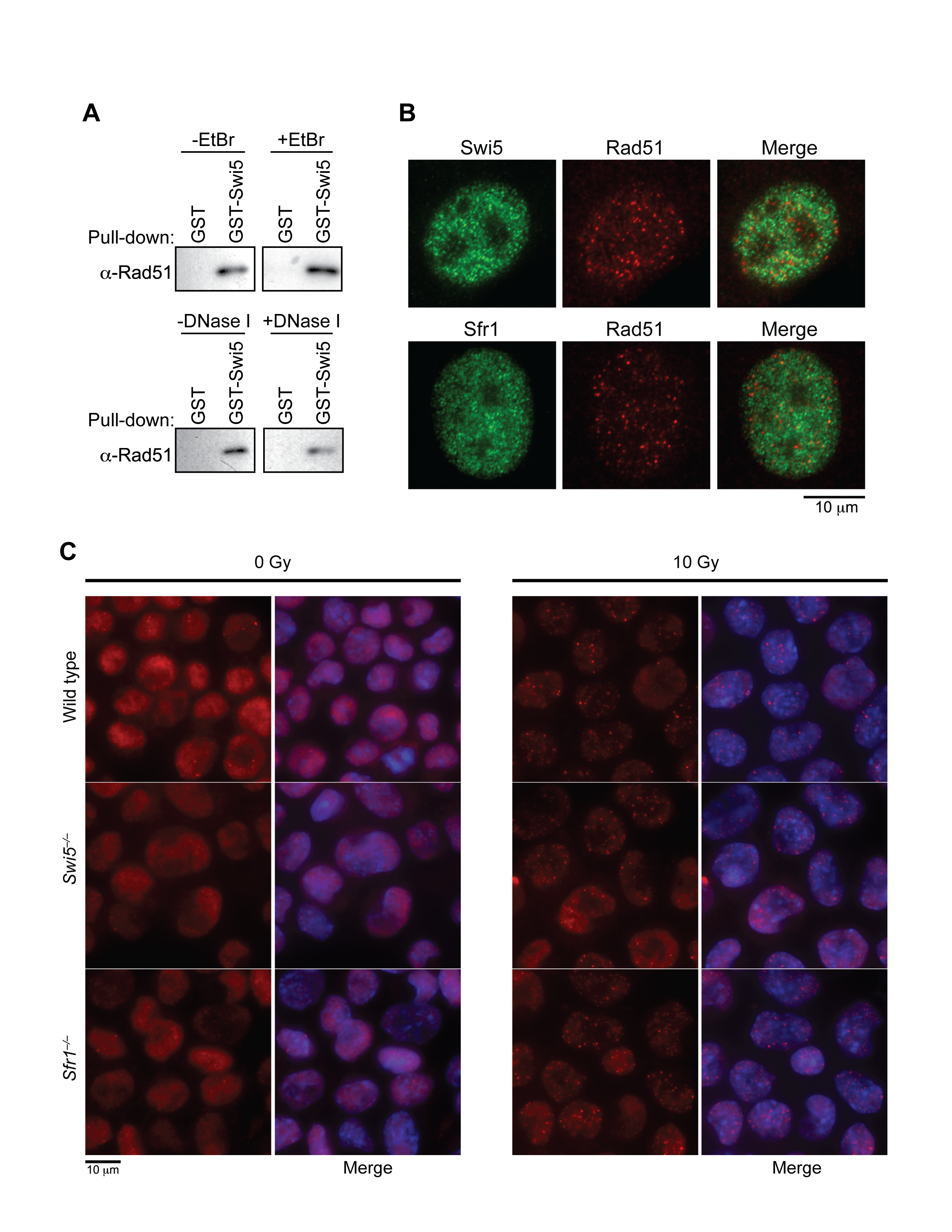

Supplement: Figure S4 — (A) Ethidium bromide (EtBr) and DNase I treatments did not interfere with co-precipitation of Rad51 by GST-Swi5 in the pull-down assay. During incubation of GST-Swi5 and Rad51 protein, 0.3 mg/ml of EtBr was added to the reaction. The DNase I treatment was performed against precipitates of GST or GST-Swi5 with 10 U of DNase I for 15 min at room temperature. After adding EDTA to stop the reaction, the precipitates were washed three times with TE and eluted with SDS-PAGE sample buffer. (B) Staining of Rad51 with Swi5 or Sfr1 in MEF cells observed 3 hours after 8 Gy of X-irradiation. (C) Rad51 focus formation was not affected in Swi5−/− and Sfr1−/− cells. The indicated mouse ES cell lines were exposed to 10 Gy of X-irradiation. Four hours post irradiation more than 90% of Swi5−/−and Sfr1−/− cells formed discrete Rad51 foci as similarly observed in wild-type cells. The merge images show co-staining with DAPI. (9.18 MB TIF) [file pgen.1001160.s004.tif]

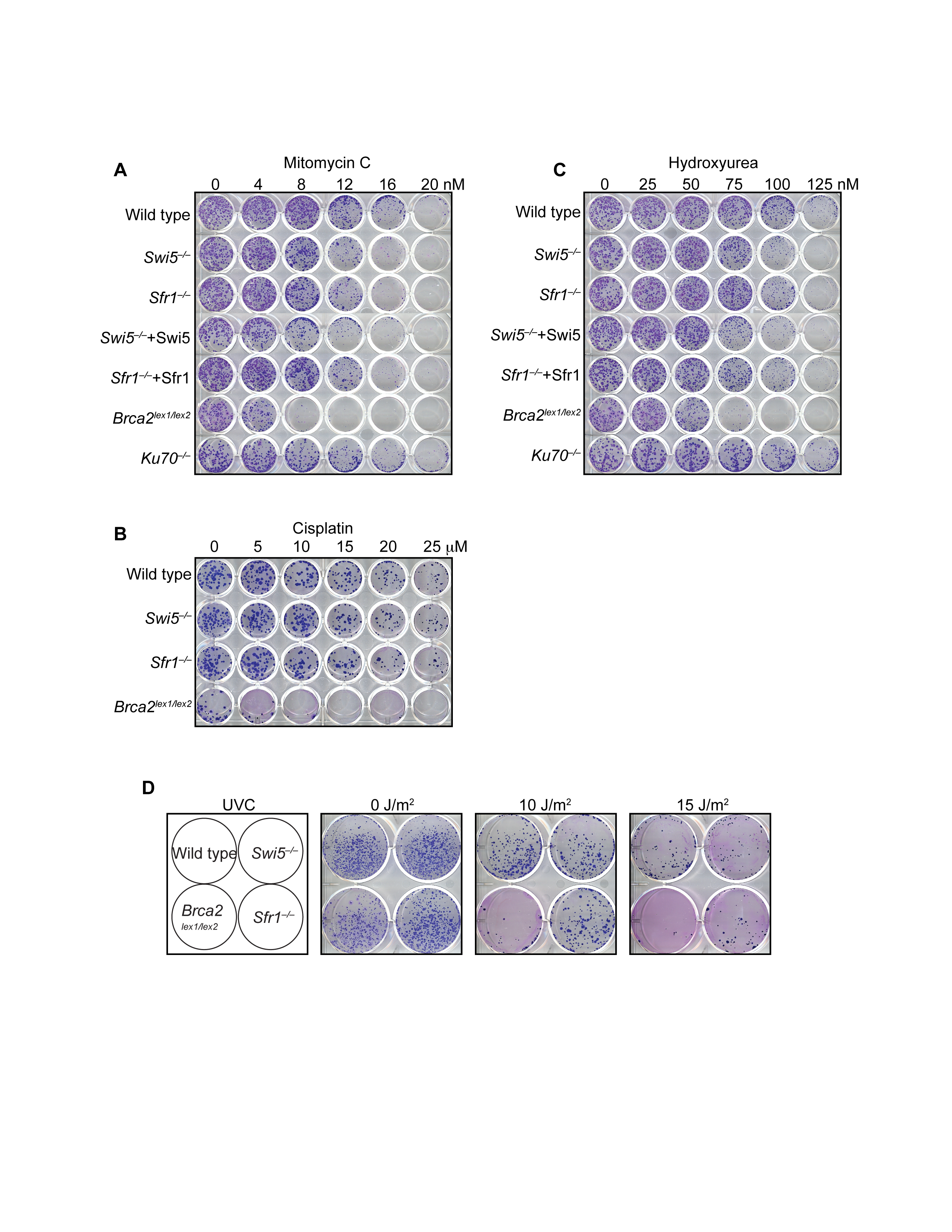

Supplement: Figure S5 — Swi5−/−and Sfr1−/− cells were not sensitive to mitomycin C (A), cisplatin (B), hydroxyurea (C) or UVC (D). Cells, seeded at 500 cells per well of a 24-well plate 24 hours earlier, were exposed to the reagents at the indicated concentrations, continuously for mitomycin C and hydroxyurea treatments and for 1 hour followed by a media change for cisplatin. Cells irradiated with UVC were seeded at 5000 cells per well of a 6-well plate 24 hours in advance of exposure at the indicated dose. After 7 days of incubation, cells were fixed and stained with Giemsa. (5.92 MB TIF) [file pgen.1001160.s005.tif]
