# Supplementary material for: Role for the Mammalian Swi5-Sfr1 Complex in DNA Strand Break Repair through Homologous Recombination
Source: PLoS Genet. 2010 Oct 14;6(10):e1001160. doi: 10.1371/journal.pgen.1001160 (PMC2954829; doi:10.1371/journal.pgen.1001160)
Supplement: Table S1 — Chromosome aberrations with or without Parp inhibition (0.6 µM). (0.05 MB PDF) [file pgen.1001160.s006.pdf]

Table S1. Chromosome aberrations with or without Parp inhibition (0.6 μM).

Genotype	Olaparib	Number				
		Cells	Chromosomes	Chromatid breaks/gaps	Radial chromosomes	Total aberrations
Wild-type	–	34	1367	2	0	2
	+	51	2045	23	4	27
Swi5$^{-/-}$	–	69	2743	5	0	5
	+	40	1595	74	12	86
Sfr1$^{-/-}$	–	60	2387	8	0	8
	+	51	2041	127	9	136
Brca2$^{lex1/lex2}$	–	66	2638	36	2	38
	+	64	2502	132	19	151
